# Minocycline-Derived Silver Nanoparticles for Assessment of Their Antidiabetic Potential against Alloxan-Induced Diabetic Mice

**DOI:** 10.3390/pharmaceutics13101678

**Published:** 2021-10-14

**Authors:** Syed Akif Raza Kazmi, Muhammad Zahid Qureshi, Sadia, Saleh S. Alhewairini, Shaukat Ali, Shazia Khurshid, Muhammad Saeed, Shumaila Mumtaz, Tafail Akbar Mughal

**Affiliations:** 1 Department of Chemistry, Government College University Lahore, Lahore 54000, Pakistan; dr.zahidqureshi@gcu.edu.pk (M.Z.Q.); shaziakhurshid1@yahoo.com (S.K.); 2 Science Unit, Department of Biochemistry, Deanship of Educational Services, Qassim University, Buraidah 51452, Saudi Arabia; 3 Department of Biotechnology, University of Azad Jammu and Kashmir, Muzaffarabad 13100, Pakistan; sadiamughal611@gmail.com; 4 Department of Plant Production and Protection, College of Agriculture and Veterinary Medicine, Qassim University, Buraidah 51452, Saudi Arabia; hoierieny@qu.edu.sa; 5 Medical Toxicology Laboratory, Department of Zoology, Government College University Lahore, Lahore 54000, Pakistan; shumaila.mzfd@gmail.com (S.M.); tufail.akbar9@gmail.com (T.A.M.); 6 Department of Chemistry and Chemical Engineering, SBASSE LUMS, Lahore 54692, Pakistan; muhammad.saeed@lums.edu.pk; 7 Department of Zoology, Women University of Azad Jammu and Kashmir, Bagh 12500, Pakistan

**Keywords:** minocycline, silver nanoparticles, tetracycline, antidiabetic, in vivo, nanomedicine

## Abstract

Diabetes is a life-threatening disease, and chronic diabetes affects parts of the body including the liver, kidney, and pancreas. The root cause of diabetes is mainly associated with oxidative stress produced by reactive oxygen species. Minocycline is a drug with a multi-substituted phenol ring and has shown excellent antioxidant activities. The objective of the present study was to investigate the antidiabetic potential of minocycline-modified silver nanoparticles (mino/AgNPs) against alloxan-induced diabetic mice. The mino/AgNPs were synthesized using minocycline as reducing and stabilizing agents. UV-visible, FT-IR, X-ray diffraction (XRD), and transmission electron microscopy (TEM) were applied for the characterization of mino/AgNPs. A 2,2-diphenyl-1-picrylhydrazyl free radical scavenging assay was conducted to determine the antioxidant potential of newly synthesized mino/AgNPs. The results revealed that the mino/AgNPs showed higher radical scavenging activity (IC_50_ = 19.7 µg/mL) compared to the minocycline (IC_50_ = 26.0 µg/mL) and ascorbic acid (IC_50_ = 25.2 µg/mL). Further, mino/AgNPs were successfully employed to examine their antidiabetic potential against alloxan-induced diabetic mice. Hematological results showed that the mice treated with mino/AgNPs demonstrated a significant decrease in fasting blood glucose level and lipid profile compared to the untreated diabetic group. A histopathological examination confirmed that the diabetic mice treated with mino/AgNPs showed significant recovery and revival of the histo-morphology of the kidney, central vein of the liver, and islet cells of the pancreas compared to the untreated diabetic mice. Hence, mino/AgNPs have good antidiabetic potential and could be an appropriate nanomedicine to prevent the development of diabetes.

## 1. Introduction

Diabetes mellitus, along with its secondary complications, continue to be a major threat to human health all over the world [1]. It is one of the five main causes of death globally [2]. It is a group of diseases that occur as a consequence of hyperglycemia and glucose intolerance, known as diabetes mellitus (DM). Two types of diabetes mellitus are conventionally known. Insufficient secretion of the hormone insulin from β-cells of the pancreas is classified as type-1 DM, and the development of insulin resistance in the body is classified as type-2 DM [3]. Globally more than 90% of diabetes patients suffer from type-2 DM [4]. Alarmingly, the numbers are increasing at a dreadful rate. According to Veiseh et al., more than 280 million adults are suffering from diabetes mellitus, and the high prevalence of DM may cause the 400 million adults to be affected till 2030 [5]. 

The root cause of diabetes is mainly associated with oxidative stress produced by reactive oxygen species (ROS) that induce β-cells dysfunction, insulin resistance, and impaired glucose tolerance. Excess food and lack of physical activity contribute to the overload of glucose and fatty acids that leads to the formation of ROS [6]. According to Rohdes, the pancreatic β-cells are highly sensitive to physiological and pathological stressors, resulting in a loss of insulin, triggered by apoptotic cell death [7]. The studies of Volpe et al. also reported the effect of oxidative stress on pancreatic β-cell death and associated diabetic complications. According to them, diabetes-associated complications that are induced by hyperglycemia are mainly because of an imbalance between ROS, which leads to higher oxidative stress and cellular death [8]. Thus, these diabetic complications can effectively be controlled by down-regulating the generation of ROS.

The change of lifestyle, diet, and oral administration of antidiabetic agents are the key factors to down-regulate the generation of ROS regarding the treatment of diabetes [5,6]. The primary objective for both type-1 DM and type-2 DM is maintaining the persistent control of glucose level within the normal glycemic range (70–140 mg/dL) [5]. The selection of a suitable drug is a common problem in the treatment of diabetes. Various antidiabetic drugs and hypoglycemic agents have been introduced for the treatment of diabetes, such as sulfonylureas and biguanides, but these drugs do not provide persistent control over the blood glucose level. In addition, the prolonged use of these drugs induces toxicity and undesirable adverse effects, such as gastrointestinal discomfort, hypoglycemia, pancreatic degeneration, and liver impairment in the body, which renders them less common [9]. Therefore finding new drugs and hypoglycemic agents with minimal side effects and higher efficacy is interesting and the focus of prior research elsewhere.

Nanobiotechnology is among the demanding areas of research that make use of biological substances at nanoscale and find applications for them in different fields, such as biosensors [10], diagnostics [11], bio-imaging [12], catalysis [13], drug delivery systems [14], and nanomedicine [15]. In comparison to conventional drug formulations, nanomedicine has received more attention in the last few years due to its benefits, such as more precise diagnosis, a higher percentage of recovery, and more effective therapies [16]. In particular, silver nanoparticles (AgNPs) have become more desirable in the field of nanomedicine because of their fascinating properties, such as ease of synthesis, colloidal stability, biocompatibility, bioavailability, low toxicity, and the capacity for surface modification [17,18,19]. Previous studies have reported the administration route and bioavailability of AgNPs in an animal model. Small-sized AgNPs could be easily absorbed into the gastrointestinal tract and released into the bloodstream, followed by excretion from the body via feces and urine [20]. Park et al. reported the bioavailability and excretion of citrate-coated AgNPs with an average size of 7.9 nm. According to them, the bioavailability of rats administrated orally with 1 mg/kg AgNPs was 1.2% and 4.2% in the rats exposed to the 10 mg/kg AgNPs [18]. 

AgNPs can reduce the oxidative stress caused by the imbalance between reactive oxygen species (ROS). The 2,2-diphenyl-1-picrylhydrazyl (DPPH) free radical scavenging activity of AgNPs has been reported by many workers [21,22,23,24,25]. H_2_O_2_ is an important metabolic signal for the glucose-stimulated secretion of insulin from β-cells [26], whereas excessive generation of H_2_O_2_ can be harmful for the integrity and function of β-cells [27]. The studies of Campoy et al. demonstrated the use of *Eysenhardtia polystachya* silver nanoparticles for the protection of INS-I cells from H_2_O_2_-induced oxidative injury. They used an *Eysenhardtia polystachya* extract to synthesize AgNPs. They reported that the cells that were exposed to H_2_O_2_ showed marked inhibition in insulin secretion, whereas the cells that were treated with EP/AgNPs before exposure to H_2_O_2_ showed a significant increase in insulin secretion. They anticipated that the polyphenolic compounds present in *Eysenhardtia polystachya* may protect the insulin-secreting cells from oxidative stress [28]. Several studies have been published reporting the possible mechanisms for antioxidant properties of AgNPs. However, it is necessary to note that the antioxidant potential of AgNPs largely depends on the chemical composition of the compound with which it is modified. The nanoparticles prepared using extracts rich in phenolic compounds and flavonoids showed high scavenging activities [21,29].

Therefore, minocycline was selected to synthesize AgNPs. Minocycline is a semi-synthetic antibiotic from the tetracycline group. It has been used for more than 30 years as a drug of choice for the treatment of diseases related to bacterial infections. Nowadays, non-antibiotic characteristics of minocycline, such as anti-tumor, anti-inflammatory, and antioxidant [30,31] properties, have attracted the attention of scientists towards this second-generation antibiotic. Minocycline has a phenolic structure with multiple ionizable functional groups. At C_4_ carbon, minocycline has a dimethylamino group that is mainly responsible for the enhanced antioxidant potential of minocycline [32]. Lee et al. reported the antioxidant activities of minocycline against the oxidative stressor (H_2_O_2_). According to them, flies treated with minocycline showed more resistance to hydrogen peroxide (H_2_O_2_) and died less often compared to the flies that did not receive minocycline treatment [33]. Murakami et al. also reported the free radicals scavenging activity of minocycline. The study demonstrated that the antioxidant activity of minocycline is 200 to 300 times more effective than that of tetracycline. According to them, minocycline is a chain-breaking antioxidant with antioxidant activities comparable to that of Trolox and α-tocopherol [32]. Several previous reports have been published demonstrating that minocycline is an effective antioxidant with free radical scavenging potency similar to vitamin C and E [34]. 

Considering the antioxidant potential of minocycline and AgNPs, the present study was aimed to check the antidiabetic potential of mino/AgNPs against alloxan-induced diabetic mice. The mino/AgNPs were synthesized and extensively characterized using UV-vis., X-ray diffraction (XRD), FT-IR, and transmission electron microscopy (TEM). Then, the synthesized mino/AgNPs were successfully applied to examine their in vivo antidiabetic potential against alloxan-induced diabetic mice. 

## 2. Experiment

### 2.1. Ethical Statement

All animal trial techniques were directed as per local and worldwide controls. These techniques come from the Wet op de dierproeven (Article 9) of Dutch Law (International) as detailed in our previous studies [35,36,37,38,39,40,41,42,43] and The Institutional Bioethics Committee at Government College University Lahore, Pakistan (No. GCU/IIB/21 dated: 08-01-2019).

### 2.2. Materials

Silver nitrate, minocycline, alloxan monohydrate, and sodium chloride were purchased from Sigma-Aldrich (St. Louis, MO, USA). Sodium hydroxide was purchased from Fluka Chemicals (Milwaukee, WI, USA). The other chemicals were of analytical grade and used without further purification.

### 2.3. Synthesis of Minocycline-Derived Silver Nanoparticles 

The mino/AgNPs were synthesized using minocycline as a reducing and stabilizing agent. A total of 2 mL silver nitrate (0.8 mM) and 2 mL (0.8 mM) minocycline solution were placed in a conical flask. To the mixture, a few drops of 0.01 M sodium hydroxide were added to accelerate the synthesis process. The resulting mixture was continuously stirred for 4 min. A UV-visible spectrophotometer (UV-1700, Shimadzu, Kyoto, Japan) was used to monitor the synthesis of mino/AgNPs in the wavelength range 300–800 nm.

### 2.4. Characterization of the Minocycline-Modified Silver Nanoparticles

The size and morphology of prepared mino/AgNPs were determined using a transmission electron microscope (TEM) using an FEI Tecnai t12 running at 80 kV with final emission of about 10 µA. A 2 k AMT camera was used to take micrographs. The sample was produced on a copper grid with carbon coating and formvar film, dropped 10 µL of mino/AgNPs solution, and was then dry overnight and analyzed using TEM. Furthermore, the colloidal stability of as-synthesized mino/AgNPs was examined by measuring their zeta potential using a zeta sizer (Malvern Instruments, Worcestershire, UK) at 25 °C.

The role of minocycline in the synthesis of mino/AgNPs was examined through FT-IR analysis. To prepare the sample for FT-IR analysis, a colloidal mino/AgNPs solution was centrifuged three times at 10,000 rpm for 30 min and washed each time with deionized water. The pellet obtained after centrifugation was left overnight to dry under the fume hood. FT-IR analysis was carried out with Bruker Alpha (Ettlingen, Germany).

The crystalline nature of prepared mino/AgNPs was studied using XRD. To prepare the sample for XRD studies, a colloidal solution of mino/AgNPs was centrifuged three times at 10,000 rpm for 30 min and washed with deionized water each time. Then, the pellet obtained after centrifugation was left overnight to dry under the fume hood. XRD was carried out in the 2θ region, from 0 to 80° with a BRUKER D2 PHASER (Karlsruhe, Germany). The scanning rate was 0.02° per minute. The Cu K_α1_ radiation having wavelength (λ) 1.5406 Å was utilized along with 40 mA tube current and 40 kV tube voltages.

### 2.5. Antioxidant Study—DPPH Assay

The antioxidant potential of minocycline, mino/AgNPs, and ascorbic acid were evaluated through DPPH free radical scavenging assay. Briefly, 100 µL of each minocycline, mino/AgNPs, and ascorbic acid at various concentrations (10, 25, 50, and 100 µg/mL) were added to the 2.9 mL of 0.1 mM DPPH solution in methanol. The resulting mixtures were kept in dark for 30 min. The DPPH solution (2.9 mL DPPH and 100 µL methanol) was used as a control solution. The absorbance of control and reaction mixtures was measured at 517 nm using a UV-vis spectrophotometer (UV-1700, Shimadzu, Kyoto, Japan). The DPPH scavenging activity was expressed as a percentage and was calculated by the following formula:

(1) $$DPPH\;Scavenging\;Effect\left(\%\right)=\frac{Absorbance\;of\;Control\;-Absorbance\;of\;Sample}{Absorbance\;of\;Control}\;\times 100$$

### 2.6. Experimental Animals

Thirty-two albino mice were received from the University of Veterinary and Animal Sciences. Before any kind of experimentation, the mice were left in the animal house for two weeks at 25 °C with frequent access to water and food. This was done to acclimatize the mice to a new environment. The weight of the bodies of all of the mice was measured before and after the treatment. All of the experiments performed during the in vivo studies were approved by the Bioethical Committee of Government College University Lahore, Pakistan. 

### 2.7. Induction of Diabetes

Alloxan monohydrate is a toxic glucose analog that affects the β-cells of the pancreas and is frequently used in an animal model to induce diabetes [44]. The intraperitoneal injection of alloxan monohydrate (100 mg/Kg body weight) resulted in the induction of diabetes to the overnight fasted mice. Subsequently, to protect the mice from hypoglycemic effects, they were fed with glucose solution (10%) for 24 h along with normal food. To confirm the induction of diabetes, the fasting blood sugar level of mice was measured regularly with 3 day intervals, up to 14 days. Those mice were considered diabetic and were selected for further experimentation if they had fasting blood sugar of more than 250 mg/dL.

### 2.8. Experimental Design

To conduct the antidiabetic studies, four groups were created, with eight mice in each group. Group-I; normal control, group-II; diabetic left untreated, group-III; diabetic treated with the drug glibenclamide (5 mg/Kg body weight), group-IV; diabetic treated with mino/AgNPs (5 mg/Kg body weight). The mice were treated regularly for 28 days with glibenclamide and mino/AgNPs via oral administration.

### 2.9. Collection of Sample

After the successful completion of twenty-eight days of treatment, the mice fasted for 12 h, and subsequently, they were given anesthesia with chloroform. The mice were then dissected sacrificially and blood samples were obtained by heart puncture in three distinct tubes. The kidney, pancreas, and liver were dissected, followed by washing with phosphate buffer saline (to clear debris) and placed in a 10% formalin solution for further processing.

### 2.10. Biochemical Assay

Blood sugar level and hemoglobin were measured using commercially available kits. Serum lipid profiles such as triglycerides and total cholesterol were estimated using respective kits from BD Biosciences, San Jose, CA, USA. Serum glutamic oxaloacetic transaminase (SGOT) and serum glutamate pyruvate transaminase (SGPT) were determined using a standard international federation of clinical chemistry (IFCC) kinetic method (BD Biosciences, San Jose, CA, USA).

### 2.11. Histopathological Studies

For overnight fixation, the kidney, liver, and pancreas were added in the 10% formalin solution. Afterward, the dehydration of slices (3–4 mm) of the liver, kidney, and pancreas tissues was performed using ascending grades of alcohol, then cleared (alcohol was extracted) with xylene and embedded in paraffin wax (58–60 °C). Blocks were made and sectioned of 5 mm thickness with a microtome. The staining of tissue sections was done with hematoxylin and eosin staining [45]. The light microscope was used for the examination of prepared slides.

### 2.12. Statistical Analysis

The statistical analysis of the data was performed through ANOVA using statistix 10 software (Analytical software, Tallahassee, FL, USA), and the data were presented as means ± standard deviation. The least significant difference (LSD) test was applied for multiple comparisons among the mean values. The differences were considered statistically significant at *p* ≤ 0.05. The figures were plotted using origin pro. 8 software (OriginLab Corporation, Northampton, MA, USA).

## 3. Results and Discussion

### 3.1. Strategy of Assay 

Diabetes mellitus with its chronic condition consistently remains a major threat to life. Down-regulating the generation of reactive oxygen species could be an alternative to reduce diabetes-associated complications. Minocycline is a semi-synthetic drug with excellent antioxidant properties similar to vitamin C. Furthermore, the synergic effects of silver nanoparticles as an antioxidant could be utilized to increase the effectiveness of such phenolic antioxidants [23]. Thus, in the present work, the minocycline-modified sliver nanoparticles (mino/AgNPs) were prepared using minocycline as a reducing and capping agent. The prepared mino/AgNPs were subjected to extensive characterization and successfully applied to examine their in vivo antidiabetic potential against alloxan-induced diabetic mice. 

### 3.2. Synthesis and Stability of Minocycline-Modified Silver Nanoparticles

**Figure 1:**
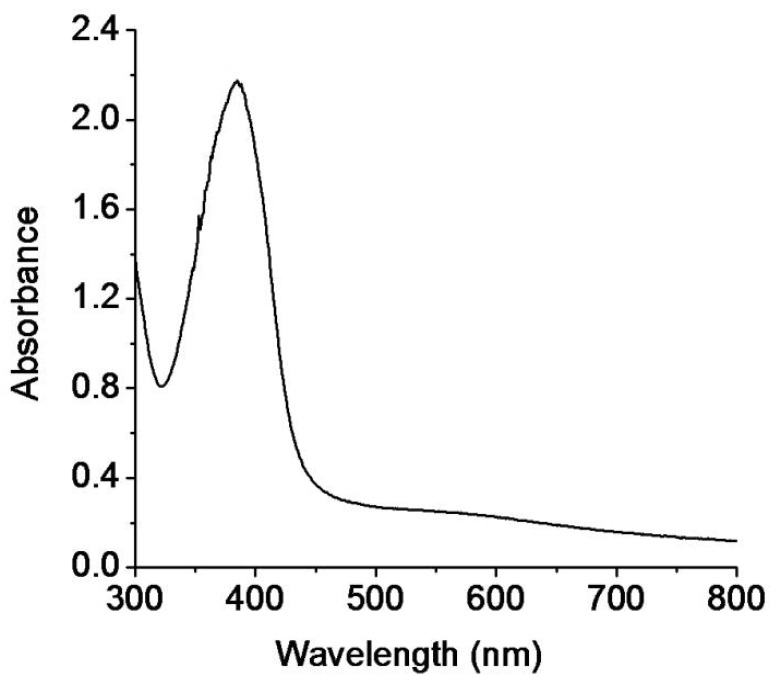
UV-visible spectrum of minocycline-modified silver nanoparticles.

**Figure 2:**
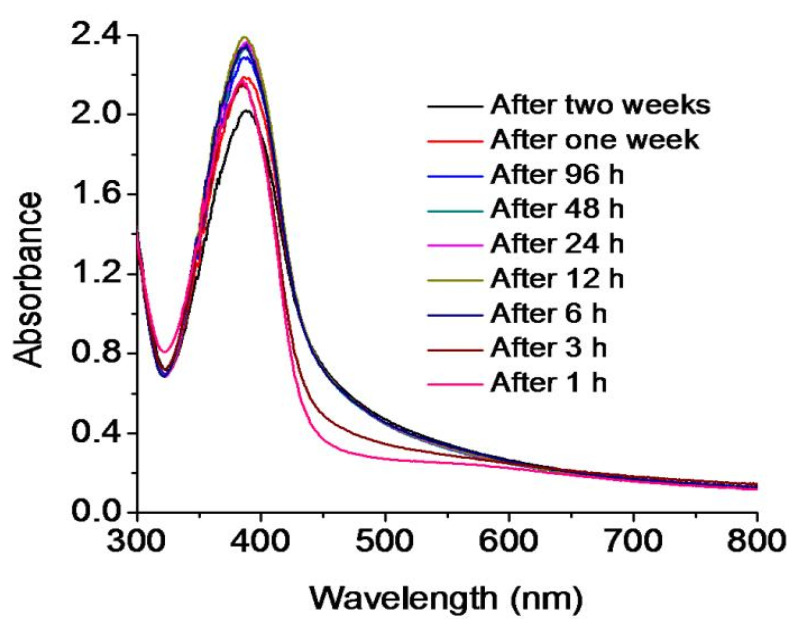
UV-visible spectra indicating the stability of mino/AgNPs.

The UV-vis. spectrophotometer is an important instrument to monitor the synthesis and stability of metal nanoparticles [46]. The mixture of silver nitrate, minocycline, and sodium hydroxide was continuously stirred for 4 min at room temperature. The solution changed its color from colorless to yellowish-brown within 4 min, which was the first indication of the synthesis of mino/AgNPs [47]. Furthermore, the synthesis of mino/AgNPs was monitored through a UV-vis. spectrophotometer (UV-1700, Shimadzu, Kyoto, Japan) in the wavelength range 300–800 nm. A sharp LSPR band was observed at 395 nm (Figure 1), which is a characteristic LSPR for silver nanoparticles [48]. The stability of mino/AgNPs was monitored for two weeks from plasmon wavelength (λmax), as aggregation causes the red-shift of their spectra [46]. No significant shift in wavelength (λmax) was observed for the LSPR of the mino/AgNPs colloidal solution until two weeks (Figure 2), which indicates the good stability of the as-synthesized mino/AgNPs. 

### 3.3. Characterization of Minocycline-Modified Silver Nanoparticles

**Figure 3:**
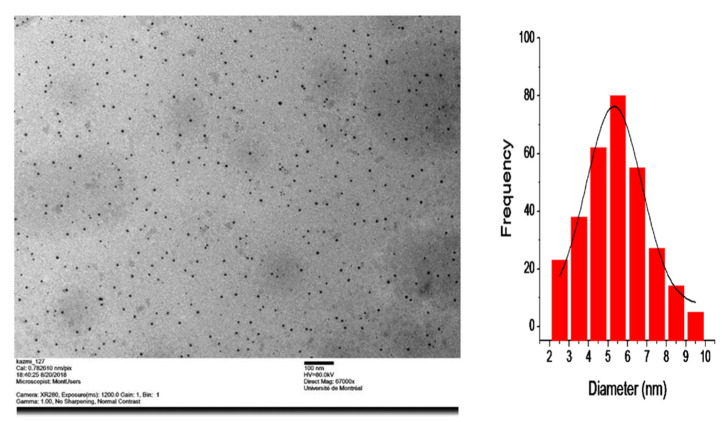
TEM and histogram of mino/AgNPs.

**Figure 4:**
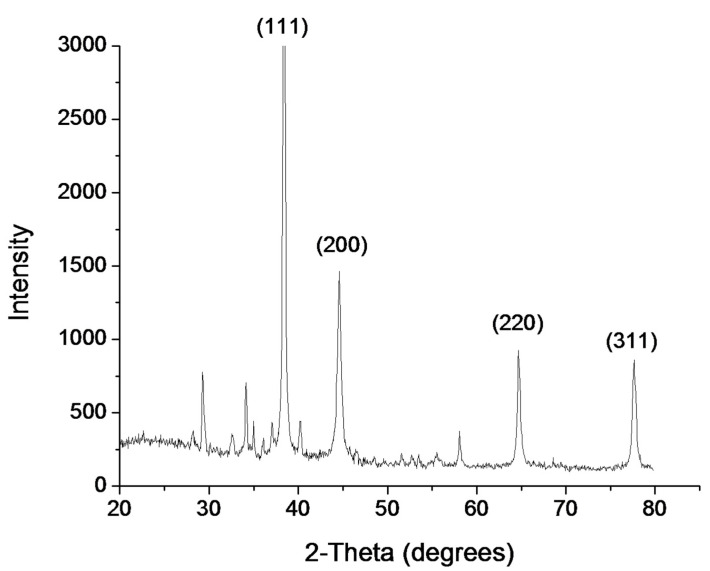
X-ray Diffraction of minocycline-modified silver nanoparticles.

The size and morphology of prepared mino/AgNPs were examined through TEM. Homogenously distributed spherical silver nanoparticles were obtained with this method (Figure 3). The average particle size of mino/AgNPs calculated was 5.5 nm. Zeta potential is a useful and important parameter to examine the stability of colloidal solution. The zeta potential of mino/AgNPs was found to be −23.4 mV, which indicates the good stability of synthesized mino/AgNPs [49]. The crystalline nature of mino/AgNPs was examined through XRD analysis. XRD pattern of mino/AgNPs showed strong diffraction peaks at 38.3, 44.5, 64.6, and 77.5 corresponding to (111), (200), (220), (311), which reflects the crystalline nature of mino/AgNPs (Figure 4). Our XRD results of mino/AgNPs are similar to the results reported elsewhere [47]. 

**Figure 5:**
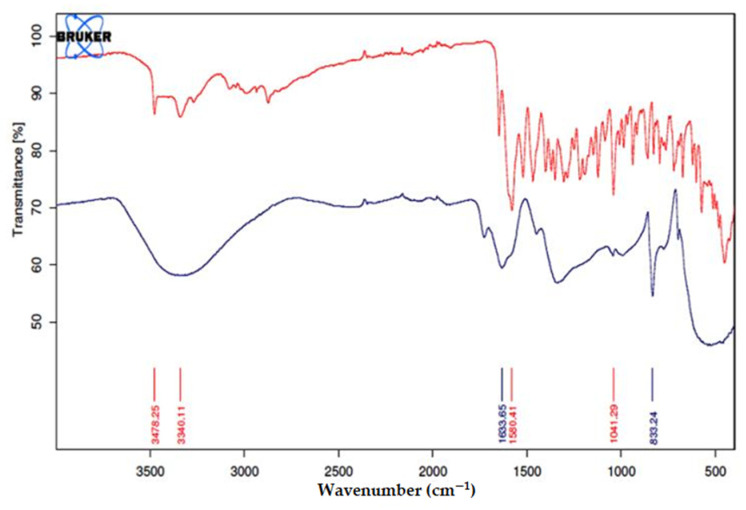
FT-IR Spectra of minocycline (Red) and minocycline-modified silver nanoparticles (Blue).

To examine the role of minocycline in the synthesis of AgNPs, the FT-IR spectra of minocycline (red) and mino/AgNPs (black) were compared (Figure 5). The spectrum of minocycline demonstrated two nominated signals at 3478.25 cm^−1^ for O-H bond stretching and 3340.11 cm^−1^ for N-H bond stretching. In addition to these two, some other signals were also present in the vicinity of these signals in the region of 3500–3100 cm^−1^, due to alkylic and vinylic alcohols. All of these signals became a single broad signal after the formation of nanoparticles, which showed the involvement of these groups in the reduction of silver ions. The broadness of the signal also showed the presence of stretched N-H bonds involved in the stabilization of nanoparticles. The signal at 1580.41 cm^−1^ was attributed to C=C stretching. The shifting of this signal to 1633.65 cm^−1^ showed the involvement of the β-hydroxy group in the reduction of silver ions and itself being oxidized to the carbonyl. The involvement of this hydroxyl group resulted in the disappearance of C=C conjugation, so the signal for amidic carbonyl appeared in a normal range. The signal for carbonyl of ketone can also be observed in the vicinity of 1580.41 cm^−1^. This signal is also shifted to a higher value like that of carbonyl of amide due to similar reasons.

### 3.4. DPPH Radical Scavenging Assay 

**Figure 6:**
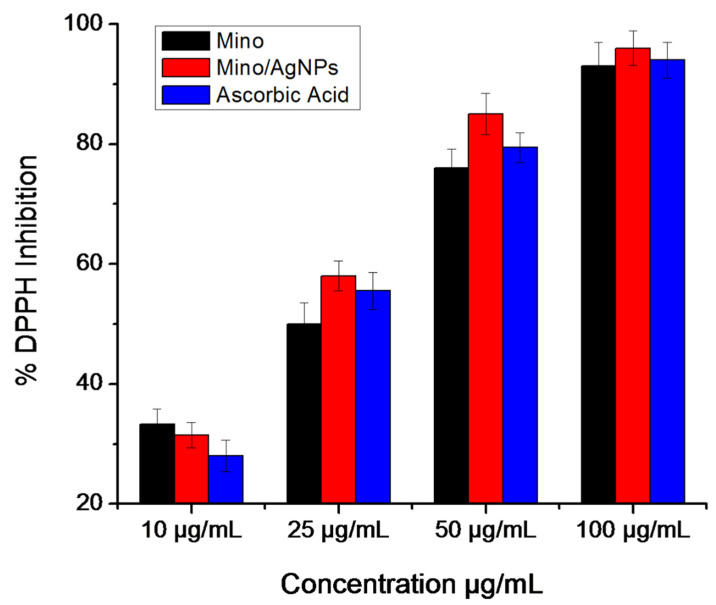
DPPH free radical scavenging assay.

The free radical scavenging activities of minocycline, mino/AgNPs, and ascorbic acid were evaluated through DPPH assay. The results demonstrated that the percentage of inhibition was concentration-dependent and in general, increased with the increase in concentrations of each analyte (Figure 6). It was observed that minocycline showed radical scavenging potency similar to that of ascorbic acid. Furthermore, the mino/AgNPs showed higher radical scavenging activity (IC_50_ = 19.7 µg/mL) compared to minocycline (IC_50_ = 26.0 µg/mL) and ascorbic acid (IC_50_ = 25.2 µg/mL). We anticipated that the increased radical scavenging activity of mino/AgNPs might be due to the synergic effects of AgNPs as antioxidant. Elemike et al. also reported the effect of AgNPs on enhancing the radical scavenging activity of *Costus afer* extract. The study reported that the *Costus afer*-modified AgNPs (CA-AgNPs) showed higher DPPH radical scavenging activities compared to the *Costus afer* leaf extract. They suggested that the increase in the antioxidant potential of CA-AgNPs can be due to the presence of phytochemicals such as flavonoids (with many hydroxyl groups) on the surface of AgNPs that contributed to the proceeding antioxidant activities through the hydrogen atom transfer (HAT) and single electron transfer (SET) mechanisms simultaneously [22]. 

### 3.5. Antihyperglycemic Activity of Minocycline-Modified Silver Nanoparticles in Alloxan-Induced Diabetic Mice 

**Figure 7:**
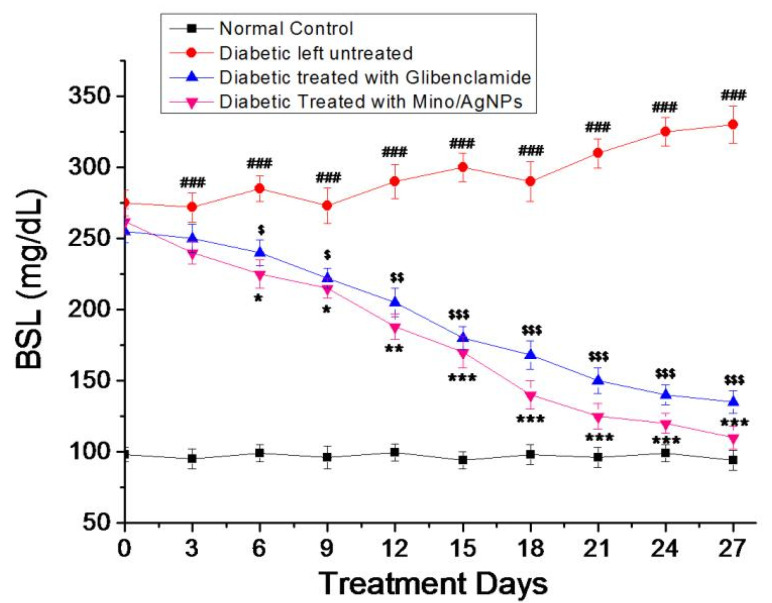
Level of blood sugar (mg/dl) for various study groups. Group-I; normal control, Group-II; diabetic left untreated, Group-III; diabetic treated with glibenclamide, Group-IV; diabetic treated with mino/AgNPs. ‘#’ indicates a significant difference between the control and diabetic groups; ‘$’ indicates a significant difference between the diabetic and glibenclamide treatment groups; ‘*’ indicates a significant difference between the diabetic and mino/AgNPs treatment groups. Each bar represents the value of mean ± SD of eight replicates. Statistical icons: $, * = *p* ≤ 0.05; $$, ** = *p* ≤ 0.01; ###, $$$, *** = *p* ≤ 0.001.

Diabetic mice show the symptoms of diabetes mellitus such as hyperglycemia, weight loss, polyuria, and decreased insulin level. The administration of the standard drug glibenclamide and mino/AgNPs to the diabetic mice resulted in a change of blood sugar level (BSL), cholesterol level, triglyceride level, and hemoglobin level compared to the untreated diabetic mice. However, the mino/AgNPs showed higher antidiabetic potential compared to the drug glibenclamide. In diabetic mice, the BSL was notably high compared to the normal mice. However, the oral administration of mino/AgNPs to the diabetic mice resulted in a significant (*p* ≤ 0.05) lowering of BSL relative to the diabetic mice left untreated (Figure 7). The excellent hypoglycemic activities of mino/AgNPs could be attributed to the combined effect of both, i.e., minocycline and AgNPs. Minocycline, due to its tremendous antioxidant potential, could act by decreasing oxidative stress due to ROS, whereas AgNPs in addition to its antioxidant effect could act by enhancing glucose-stimulated insulin secretion through inhibiting the activity of the inhibitory enzyme dipeptidyl peptidase IV (DPP-IV) [50,51]. The combined effect of mino/AgNPs resulted in reduced oxidative stress with higher insulin secretion. As a consequence of reduced oxidative stress, insulin sensitivity also improved and thereby, increased the cellular uptake of glucose from the bloodstream and thus, down-regulated the blood sugar level in treated mice. Hurrle and Hsu also reported a similar effect of antioxidants on oxidative stress and insulin resistance. The study reported the effect of ROS on different pathways in insulin receptor signal transduction that ultimately disrupt the expression of glucose transporter 4 (GULT4), a major glucose transporter in the cell. This affects the uptake of glucose from the blood into the cell, which causes insulin resistance. However, the use of antioxidants reduces the oxidative stress that ultimately leads to the down-regulation of BSL in the bloodstream by improving insulin sensitivity [52].

**Figure 8:**
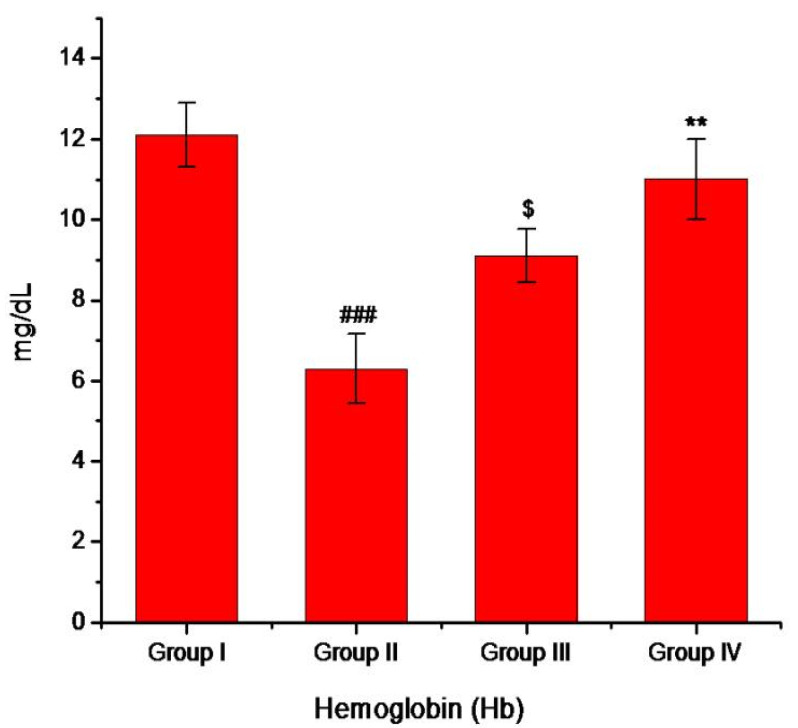
Level of hemoglobin (mg/dl) for the various study groups. Group-I; normal control, Group-II; diabetic left untreated, Group-III; diabetic treated with glibenclamide, Group-IV; diabetic treated with mino/AgNPs. ‘#’ indicates a significant difference between the control and diabetic groups; ‘$’ indicates a significant difference between the diabetic and glibenclamide treatment groups; ‘*’ indicates a significant difference between the diabetic and mino/AgNPs treatment groups. Each bar represents the value of mean ± SD of eight replicates. Statistical icons: $ = *p* ≤ 0.05; ** = *p* ≤ 0.01; ### = *p* ≤ 0.001.

Furthermore, both more weight loss and a decrease in total hemoglobin level were also observed in diabetic mice compared to the normal mice. In diabetic conditions, the reaction between excess glucose and hemoglobin converts hemoglobin to glycosylated hemoglobin as a result of which the level of hemoglobin decreases in diabetic mice [4]. However, the treatment of diabetic mice with mino/AgNPs resulted in a significant (*p* ≤ 0.05) increase in body weight (Table 1) and hemoglobin level compared to the untreated diabetic mice (Figure 8).

**Figure 9:**
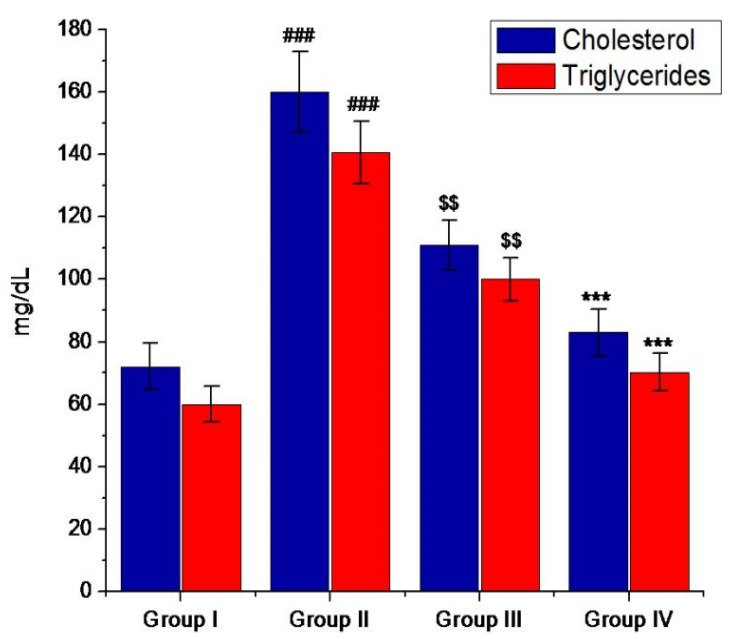
Levels of lipids (mg/dl) of various study groups. Group-I; normal control, Group-II; diabetic left untreated, Group-III; diabetic treated with glibenclamide, Group-IV; diabetic treated with mino/AgNPs. ‘#’ indicates a significant difference between the control and diabetic groups; ‘$’ indicates a significant difference between the diabetic and glibenclamide treatment groups; ‘*’ indicates a significant difference between the diabetic and mino/AgNPs treatment groups. Each bar represents the value of mean ± SD of eight replicates. Statistical icons: $$ = *p* ≤ 0.01; ###, *** = *p* ≤ 0.001.

Lipids have a very crucial role in the progression of DM. In a diabetic condition, the serum lipid level is generally increased, which indicates a risk of coronary heart disease [53]. Hypercholesterolemia and hypertriglyceridemia are the main risk factors that can cause atherosclerosis as well as coronary heart disease, two of the secondary complications associated with DM [54]. The use of dietary or drug treatment seems to be effective in decreasing the lipid level in serum and consequently minimizing the risk of cardiovascular disease. In the present investigation, the cholesterol and triglycerides (TG) levels were notably high in diabetic mice. However, the oral administration of mino/AgNPs to the diabetic mice resulted in a significant (*p* ≤ 0.05) decrease in triglycerides and cholesterol levels as compared to the untreated diabetic mice (Figure 9). We anticipated that the increased level of ROS interferes with cell function, alters the cholesterol and triglyceride metabolism, and thus, results in higher TC and TG level. However, mino/AgNPs treatment resulted in decreased oxidative stress by scavenging free radicals and ROS, the normalization of cell function, and consequently, the down-regulation of TC and TG levels [32,55,56]. 

**Figure 10:**
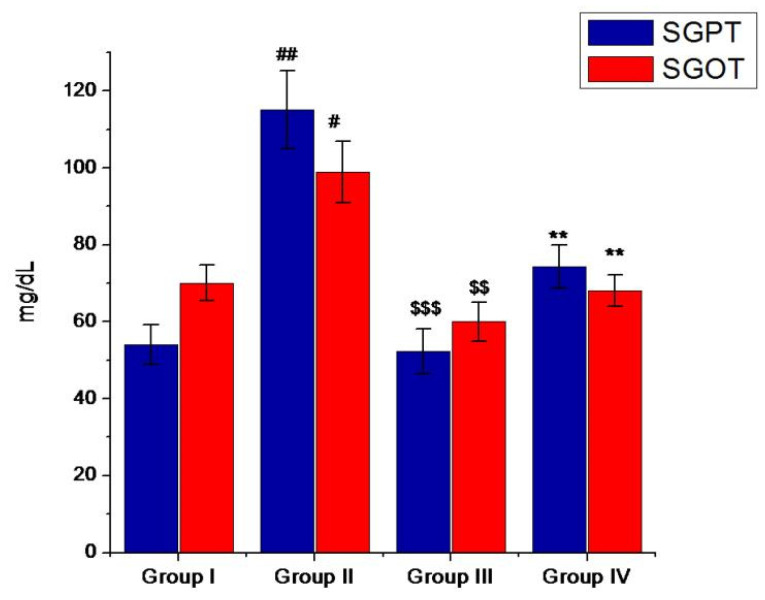
Levels of SGPT and SGOT (mg/dl) of various study groups. Group-I; normal control, Group-II; diabetic left untreated, Group-III; diabetic treated with glibenclamide, Group-IV; diabetic treated with mino/AgNPs. ‘#’ indicates a significant difference between the control and diabetic groups; ‘$’ indicates a significant difference between the diabetic and glibenclamide treatment groups; ‘*’ indicates a significant difference between the diabetic and mino/AgNPs treatment groups. Each bar represents the value of mean ± SD of eight replicates. Statistical icons: # = *p* ≤ 0.05; ##, $$, ** = *p* ≤ 0.01; $$$ = *p* ≤ 0.001.

Furthermore, the administration of mino/AgNPs to the diabetic mice also affected the activity of hepatic marker enzymes in serum. In diabetic mice, the levels of SGOT and SGPT were raised. In diabetic conditions, the liver cells are damaged, which causes the microsomal cells of the liver to excrete various enzymes, such as SGOT, SGPT, and ALP [57]. However, the oral administration of mino/AgNPs to the diabetic mice maintained a significantly (*p* ≤ 0.05) lower SGOT and SGPT compared to the diabetic mice left untreated (Figure 10).

### 3.6. Histology Studies

**Figure 11:**
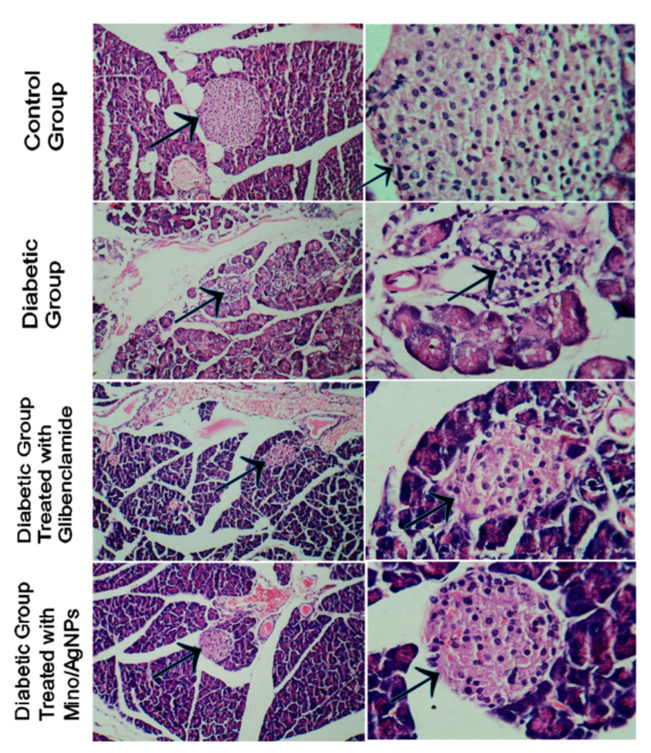
Histopathology of the islet cells of pancreatic sections of various study groups (arrowhead pointing towards the islet tissue of pancreas).

In histopathological studies, pancreas, kidney, and liver sections of treated, untreated, and normal control mice were examined. The pancreatic islet tissue of diabetic mice displayed irregular islet boundaries as well as the mass distribution of cytoplasm relative to the normal mice (Figure 11). The treatment of diabetic mice with both glibenclamide and mino/AgNPs displayed good regeneration and the recovery of the islet tissue of the pancreas. Nevertheless, mino/AgNPs showed more effectiveness in the regeneration and recovery of islet tissue than glibenclamide. The β-cell mass was significantly higher in mice treated with mino/AgNPs compared to the diabetic mice left untreated (Figure 11). We anticipated that the mino/AgNPs protected the β-cells of the pancreas from ROS and suppressed apoptosis in β-cells. The studies of Kaneto et al. also reported that the apoptosis induced by ROS in the β-cells of the pancreas was suppressed by the use of antioxidants [58].

**Figure 12:**
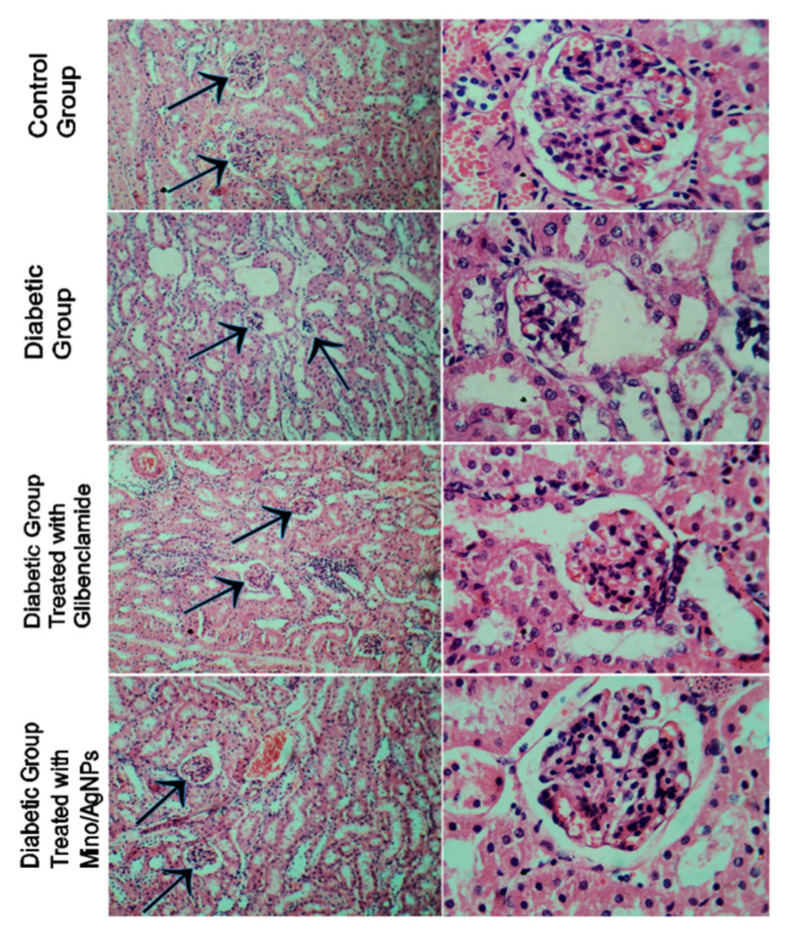
Histopathology of the kidney sections of various study groups (arrowhead pointing towards the glomerulus and urinary space of kidney).

The tissue of the normal kidney section presented the normal architecture. The kidney section of diabetic mice showed distorted glomerular and dilated urinary space with necrosis, vacuolation in the renal epithelial, and some tubules with apoptotic cells (Figure 12). The treatment of diabetic mice with glibenclamide displayed limited improvement in the morphology of glomerular with some dilated urinary space whereas the treatment of diabetic mice with mino/AgNPs displayed higher recovery and regeneration relative to the histo-morphology of the kidney sections of untreated diabetic mice. The kidney sections of diabetic mice treated with mino/AgNPs showed improved glomerular with improved urinary space very close to the architecture of normal mice (Figure 12). 

**Figure 13:**
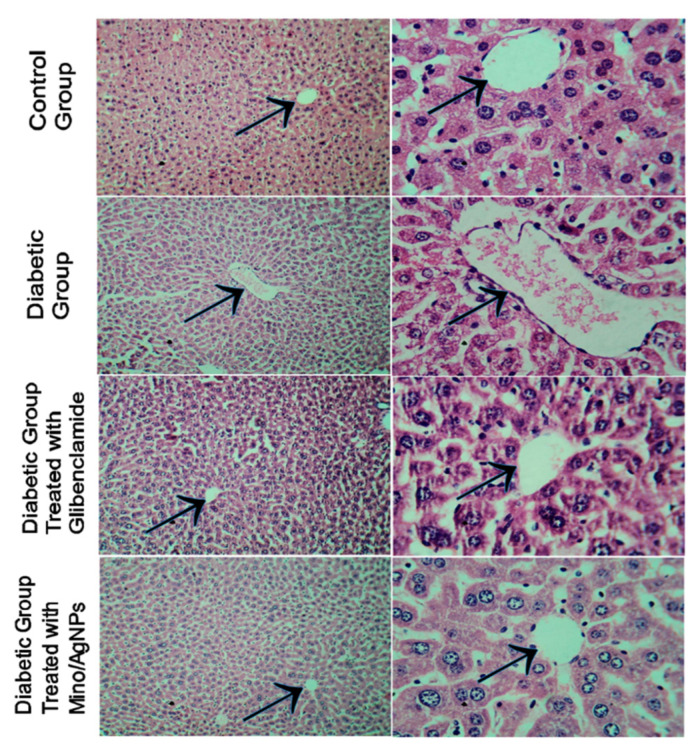
Histopathology of liver sections of various study groups (arrowhead pointing towards the central hepatic vein of the liver).

The hepatic sections of the normal liver showed normal architecture with intact central hepatic vein and slit-like sinusoids and prominent nuclei (Figure 13). The liver sections of diabetic mice displayed distorted central veins, along with apoptotic nuclei. The oral administration of both the drug and mino/AgNPs to the diabetic mice showed significant recovery of the central hepatic vein. However, the treatment with mino/AgNPs showed a better recovery and revival effect as compared to the drug glibenclamide (Figure 13).

## 4. Conclusions

Diabetes mellitus is a life-threatening disease all over the world, and it demands significant efforts to be treated effectively. Antioxidants have been shown to be very effective in many bioprocesses, including disorders of diabetes mellitus. The present work was carried out to examine the antidiabetic potential of newly synthesized mino/AgNPs in alloxan-induced diabetic mice. A DPPH inhibitory assay was conducted to compare the antioxidant potential of mino/AgNPs with that of minocycline and ascorbic acid. The mino/AgNPs showed higher radical scavenging activity (IC_50_ = 19.7 µg/mL) compared to minocycline (IC_50_ = 26.0 µg/mL) and ascorbic acid (IC_50_ = 25.2 µg/mL). Further, hematological and histopathological analysis revealed that the mino/AgNPs showed greater potential as an antidiabetic agent than the standard drug glibenclamide. The mino/AgNPs showed more effectiveness in reducing blood sugar, cholesterol, and triglycerides levels. Furthermore, the treatment of diabetic mice with mino/AgNPs also showed significant regeneration and revival of histo-morphology of the kidney, central vein of the liver, and islet cells of the pancreas compared to the untreated diabetic mice. Our results indicated that the as-synthesized mino/AgNPs have good potential to reduce the disorders of diabetes mellitus and can be effectively used to treat diabetic conditions.

## Figures and Tables

**Table 1:** Measurement of the body weight of the mice in various treatment groups.

Treatments	Groups	Weight (g) at Various Time Points during Diabetes Treatment				
		at 0 Day of Treatment	at 7th Day of Treatment	at 14th Day of Treatment	at 21st Day of Treatment	at 28th Day of Treatment
Normal Control	Group-I	34.2 ± 0.45	34.6 ± 0.61	34.8 ± 0.37	35.1 ± 0.25	35.3 ± 0.49
Diabetic	Group-II	32.8 ± 0.74	31.4 ± 0.71	29.9 ± 0.58 #	28.2 ± 0.40 ##	26.0 ± 0.35 ###
Glibenclamide	Group-III	33.0 ± 0.69	31.7 ± 0.45	32.1 ± 0.29	32.6 ± 0.18 $	32.9 ± 0.25 $$
Mino/AgNPs	Group-IV	33.4 ± 0.59	32.3 ± 0.65	32.9 ± 0.43	33.4 ± 0.27 **	33.8 ± 0.17 ***

Note: ‘#’ indicates a significant difference between the control and diabetic groups; ‘$’ indicates a significant difference between diabetic and glibenclamide treatment groups; ‘*’ indicates a significant difference between diabetic and mino/AgNPs treatment groups. Each value given in this table represents of mean ± SD of eight replicates. Statistical icons: #, $ = *p* ≤ 0.05; ##, $$, ** = *p* ≤ 0.01; ###, *** = *p* ≤ 0.001.

## Data Availability

All data are reported in this manuscript.
